# Simulation-based establishment of base pools for a hybrid breeding program in winter rapeseed

**DOI:** 10.1007/s00122-023-04519-3

**Published:** 2024-01-08

**Authors:** Daniel Krenzer, Matthias Frisch, Katrin Beckmann, Tobias Kox, Christian Flachenecker, Amine Abbadi, Rod Snowdon, Eva Herzog

**Affiliations:** 1 Department of Biometry and Population Genetics, Justus Liebig University, Giessen, Germany; 2 NPZ Innovation GmbH, Hohenlieth-Hof, Holtsee, Germany; 3 Norddeutsche Pflanzenzucht Hans-Georg Lembke KG, Hohenlieth-Hof, Holtsee, Germany; 4 Department of Plant Breeding, Justus Liebig University, Giessen, Germany

## Abstract

**Key message:**

Simulation planned pre-breeding can increase the efficiency of starting a hybrid breeding program.

**Abstract:**

Starting a hybrid breeding program commonly comprises a grouping of the initial germplasm in two pools and subsequent selection on general combining ability. Investigations on pre-breeding steps before starting the selection on general combining ability are not available. Our goals were (1) to use computer simulations on the basis of DNA markers and testcross data to plan crosses that separate genetically two initial germplasm pools of rapeseed, (2) to carry out the planned crosses, and (3) to verify experimentally the pool separation as well as the increase in testcross performance. We designed a crossing program consisting of four cycles of recombination. In each cycle, the experimentally generated material was used to plan the subsequent crossing cycle with computer simulations. After finishing the crossing program, the initially overlapping pools were clearly separated in principal coordinate plots. Doubled haploid lines derived from the material of crossing cycles 1 and 2 showed an increase in relative testcross performance for yield of about 5% per cycle. We conclude that simulation-designed pre-breeding crossing schemes, that were carried out before the general combining ability-based selection of a newly started hybrid breeding program, can save time and resources, and in addition conserve more of the initial genetic variation than a direct start of a hybrid breeding program with general combining ability-based selection.

## Introduction

Reciprocal recurrent selection (RRS) is a selection scheme that uses two pools of breeding material to increase the performance of inter-pool hybrids over breeding cycles (Melchinger and Gumber 1998). In each pool, genotypes that have the highest general combining ability (GCA, Comstock et al. 1949) to the opposite pool are selected as parental lines of the crosses, from which the base population of the next breeding cycle is derived.

As a side effect of increasing the performance of inter-pool hybrids, RRS has the consequence that the allele frequencies in the two pools diverge, and the genetic distances between the lines of the pools are increasing. The increase in allelic divergence between the pools over breeding cycles can be visualized in principal coordinate plots obtained from genotyping the inbred lines of the germplasm pools. Such diverging pools were observed, for example, in the BSSS and BSCB1 heterotic pattern of the US maize (Hagdorn et al. 2003) and in European maize breeding programs (van Inghelandt et al. 2010).

The identification of heterotic patterns by assessing the heterosis and genetic distances between groups of breeding material is often the first step in starting hybrid breeding programs. Examples include major crops such as wheat (Zhao et al. 2015; Boeven et al. 2016), rice (Beukert et al. 2017), sorghum (da Silva et al. 2020), *Brassica* species (Qian et al. 2007, 2009; Younas et al. 2012; Tian et al. 2015), maize (Reif et al. 2003a, b; Bidhendi et al. 2012; Barata and Carena 2006; Suwarno et al. 2014), and less common crops such as guava (Campos et al. 2016), triticale (Fischer et al. 2010), pearl millet (Singh and Gupta 2019; Sattler and Haussmann 2020), onions (de Souza Saraiva et al. 2022), or passion fruit (Silva et al. 2014).

Cress (1967) investigated RRS with computer simulations and found that grouping the initial germplasm to pools based on genetic distances did not result in a greater long-term response to selection compared with random assignment of the initial germplasm to the pools. Butruille et al. (2004) confirmed the results of Cress (1967) experimentally in maize. They showed that RRS for grain yield and moisture with two base populations to which the initial germplasm was randomly assigned resulted in an increase in yield and a decrease in grain moisture as well as a genetic divergence of the populations. Cowling et al. (2020) found in a simulation study on genomic selection in RRS that an initial grouping of germplasm is not required for reaching high heterosis. Summarizing, these results showed that in the long term, (a) the RRS procedure results in an increase in hybrid performance and a genetic separation of the two pools, and (b) this result is achieved independently of the initial grouping of the germplasm.

In applied breeding programs, however, not only the long-term response to selection is important, but also the fast development of experimental hybrids with high performance is essential for the economic success of the breeding program. The genetic divergence of pools that is observed in successful RRS programs suggests that there is a causal relationship between the genetic divergence and the increase in hybrid performance. For the present study, we hypothesize that such a relationship does exist. If this is the case, then pre-breeding efforts that separate breeding material genetically by planned crosses might be able to replace RRS cycles at the beginning of a hybrid breeding program. The time savings of replacing early RRS cycles by planned crosses could give the breeding program a head start and contribute to its success in providing competitive hybrids soon after the start of the breeding program. To our knowledge, there are no investigations on pre-breeding efforts that attempt to replace the first cycles of a RRS program by planned crosses that induce a genetic divergence in two germplasm pools.

Our goal was to genetically separate two pools of rapeseed germplasm, which overlap in principal coordinate plots, for their subsequent use as base pools in a hybrid breeding program. In particular, our objectives were to (1) simulate alternative crossing strategies in a three-generation crossing program for pool separation, (2) carry out the crosses that were identified as optimal for pool separation by the simulations, (3) validate the genetic separation of the two pools, and (4) compare the testcross performance of lines derived from the crossing program with that of the parental lines.

## Materials and methods

### Crossing scheme

We started the crossing program with two sets of 50 parental inbred lines, which we call pool 1 and pool 2. The lines were elite lines originating from two commercial breeding pools. Both breeding pools were adapted to central Europe, and while being managed separately, historically some exchange of material took place. Earlier crosses of lines from the two pools supported the idea that lines from these pools might complement well as parents of hybrids.

**Fig. 1:**
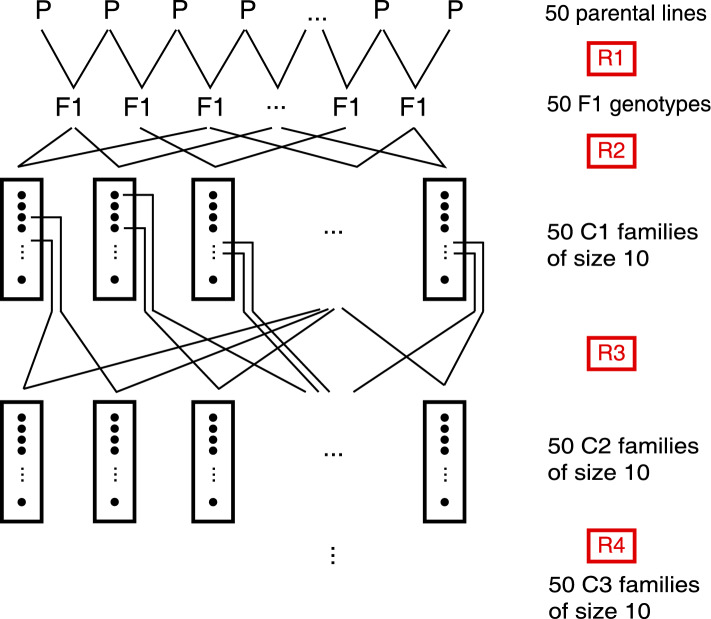
Crossing scheme for material development in one pool. Each parental line was used in two crosses, and 50 F1 genotypes were generated (recombination R1). From the F1 genotypes, 50 cycle 1 (C1) families of size 10 were generated (recombination R2). Two genotypes of each family were selected and crossed with selected genotypes from other families (recombination R3). The procedure was repeated (recombination R4) resulting in 50 C3 families of size 10

Each of the parental lines was used in two crosses to generate 50 F1 genotypes in each pool. We called this recombination step R1 (Fig. 1). Each F1 genotype was used in recombination step R2 as parent of two crosses. From each cross, a family of 10 progenies was generated. This resulted in 50 cycle 1 (C1) families of size 10. In recombination steps R3 and R4, two selected genotypes per family were crossed with the selected genotypes from two other families, and 10 progenies per cross were generated. This resulted in 50 cycle 2 (C2) and 50 cycle 3 (C3) families of size 10.

#### Crossing strategy for recombination R1

To determine which crosses of the parental lines should be carried out in recombination step R1, we investigated the target functions *T* and $T'$ that assign large values to preferable crosses. The crosses to be carried out for each of the two target functions *T* and $T'$ were determined by building sequentially a list of crosses for each target function, starting with the most preferable cross. The lines that were used for the most preferable cross were chosen such that the respective target function had the maximum value among all possible crosses. To determine the parents of the second cross in the list, all possible crosses of the two lines used in the first cross with the remaining 48 lines of the pools were compared using the respective target function. The cross with the largest value of the respective target function was added to the list of crosses. After these steps, we had identified the lines for the first cross, $\text {P1}_1\times \text {P2}_1$, and for the second cross, $\text {P1}_1\times \text {P2}_1$, where the second line of the first cross was used as the first line of the second cross. Without loss of generality, we call that parent of the *i*th cross P2_i_, which is also used in the $i+1$th cross, *i.e.*, the second crossing partner of the *i*th cross was used as the first crossing partner in the $i+1$th cross. Starting with the third cross ($i=3$), the parents P2_i_ of the cross *i* were determined such that the target function took the maximum value among all crosses of P1_i_ with the remaining lines. The last cross ($i=50$) was carried out with the lines P2_50_ and P1_1_.

The target function $T'$ was defined such that a favorable cross was characterized by two parental lines having a large modified Roger’s distance (MRD, Wright 1978, Reif et al. 2005) to the opposite pool and in addition a large MRD to each other. This is described by

1 $$\begin{aligned} T' = d(\text {P1}_i,\text {OP}) + d(\text {P2}_i,\text {OP}) + d(\text {P1}_i,\text {P2}_i), \end{aligned}$$

where $d(\text {P1}_i,\text {OP})$ is the average of the MRDs of the first parent of the cross to all lines of the opposite pool, $d(\text {P2}_i,\text {OP})$ is the average of the MRDs of the second parent of the cross to all lines of the opposite pool, and $d(\text {P1}_i,\text {P2}_i)$ is the MRD between the two parental lines.

An extension of the target function $T'$ included the MRD between the parental lines that are different in two subsequent crosses

2 $$\begin{aligned} T = d(\text {P1}_i,\text {OP}) + d(\text {P2}_i,\text {OP}) + d(\text {P1}_i,\text {P2}_i) + d(\text {P1}_{i-1},\text {P2}_i). \end{aligned}$$

Including $d(\text {P1}_{i-1},\text {P2}_i)$ avoids that the line $\text {P1}_i$ is crossed two times with two genetically similar lines with small MRD to each other. We determined the two sets of crosses that were defined by using *T* and $T'$ and compared them with respect to the similarity of the combination of lines used for the crosses.

#### Crossing strategy for recombination R2

For planning the crosses of the F1 plants, we defined two types of crosses. Type I crosses were characterized by the fact that the parental lines had a large average MRD to the genotypes of the opposite pool and a large MRD to each other. Type I crosses can be regarded as “best $\times$ best” crosses with respect to MRD to the opposite pool. The target function for type I crosses was

3 $$\begin{aligned} T_b = d(\text {P1},\text {P2})/2 + d(\text {P1},\text {OP}) + d(\text {P2},\text {OP}). \end{aligned}$$

In the target function $T_b$, the distance between the parental lines was given half the weight of the distances to the opposite pool. Each F1 genotype was used only in one cross of type I. The crosses were determined by evaluating $T_b$ of all possible crosses. One-third of the crosses was of type I. (Choosing one-third of the crosses was, after due consideration, suggested by the breeders responsible for the commercial breeding program that provided the parent pools. We plan a systematic investigation of other ratios in a future simulation study.)

Type II crosses consisted of one parent that had a large average MRD to the genotypes of opposite pool and one parent with a small distance to the opposite pool. The rationale for making type II crosses was to generate progenies that are more distant to the opposite pool than the parents that had a small MRD to the opposite pool. The type II crosses can be regarded as “improve the worst” crosses. The target function for type II crosses was

4 $$\begin{aligned} T_w = d(\text {P1},\text {P2})/2 + \max \big [ d(\text {P1},\text {OP}), d(\text {P2},\text {OP}) \big ] - \min \big [ d(\text {P1},\text {OP}), d(\text {P2},\text {OP})\big ]. \end{aligned}$$

The difference between the second and the third terms in Eq. 4 takes large values for crosses in which one parent has a large distance to the opposite pool, and the second has a small distance to the opposite pool. Two-thirds of the R2 crosses were of type II.

#### Crossing strategy for recombinations R3 and R4

For the recombinations R3 and R4, we employed a two-step procedure. In the first step, we used the 50 families as taxonomic units and estimated the MRD between families. Then, the families to be crossed were determined such that one-third of the crosses were of type I (“best $\times$ best,” Eq. 3), and two-thirds were of type II (“improve the worst,” Eq. 4).

After determining the families to be crossed, in the second step, the genotypes within the families were selected. For this step, we used testcross data for yield obtained by crossing the parental lines with a tester from the opposite pool. The testcross performance for yield was assessed in trials of an ongoing commercial breeding program. From these data, we estimated the marker effects for testcross performance using ridge-regression best linear unbiased prediction (RR-BLUP). These marker effects were then used for predicting the testcross performance of the members of each family and ranking the genotypes within a family according to testcross performance. The two best genotypes according to the ranking on basis of the estimated testcross performance were then used for crossing.

#### Genotyping and analysis of SNP data

The plant populations were genotpyed with a 15k SNP *Brassica napus* Illumina array designed by SGS Trait Genetics (Gatersleben, Germany), containing a selection of SNPs from the 60k SNP Brassica Illumina array described by Clarke et al. (2016). The genetic relationships between the genotypes of each cycle were assessed with the MRD (Wright 1978, Reif et al. 2005) and visualized with principal coordinate analyses (Gower 1966). The gene diversity (Nei 1973) was determined to measure the diversity within the two populations in each cycle.

#### Simulations

In each generation of the crossing program (Fig. 1), we used the marker genotypes of the current generation for simulating the crosses and marker genotypes of the following generation to visualize the consequences of alternative crossing decisions. The simulations were carried out step by step from one generation to the next. We compared the alternative target functions in Eqs. 1 and 2 with simulations using the marker genotypes of the 50 lines of the base pools. The split ratio of 1/3–2/3 of “best $\times$ best” (Eq. 3) and “improve the worst” (Eq. 4) was not systematically evaluated in this study, we plant further simulations on this topic.

We used our software package SelectionTools (http://population-genetics.uni-giessen.de/~software/) for carrying out the simulations.

#### Preliminary evaluation of testcross performance

For a preliminary evaluation of the success of our crossing program with respect to improving the testcross performance for yield, we sampled randomly four C1 and six C2 genotypes from pool 1, as well as two C1 genotypes and six C2 genotypes from pool 2. From these plants, 35 C1-DH lines (Weber et al. 2005) and 59 C2-DH lines were generated for pool 1 and 28 C1-DH and 54 C2-DH lines from pool 2. The DH lines were tested together with the parental lines and standards in the trials of an ongoing commercial breeding program for testcross performance using one tester from the opposite pool. The testers were lines that were used in the ongoing breeding program as testers.

The trial was carried out as a generalized lattice with two replications at five locations in 1 year. The data were analyzed with the mixed linear model $y_{ijk} = \beta _0 + g_i + u_j + gu_{ij} + r_{k(j)} + b_{l(j)}+ e_{ijk}$, where $g_i$ was the fixed effect of the hybrid. The random effects in the model were the environmental effect $u_j$, the genotype by environment interaction effect $gu_{ij}$, the replication effect nested within environment $r_{k(j)}$, and the lattice block effect within environment $b_{l(j)}$, $e_{ijk}$ was the residual error. The relative yield with respect to the three standards Ludger, DK Excited, and LG Aviron was assessed.

## Results

The SNP data were pre-processed by removing markers with more than 10% missing values and a gene diversity (Nei 1973) of less than 0.01 in the base populations. After pre-processing the data set consisted of 12,537 markers, the chromosomes contained between 240 and 1140 markers.

**Fig. 2:**
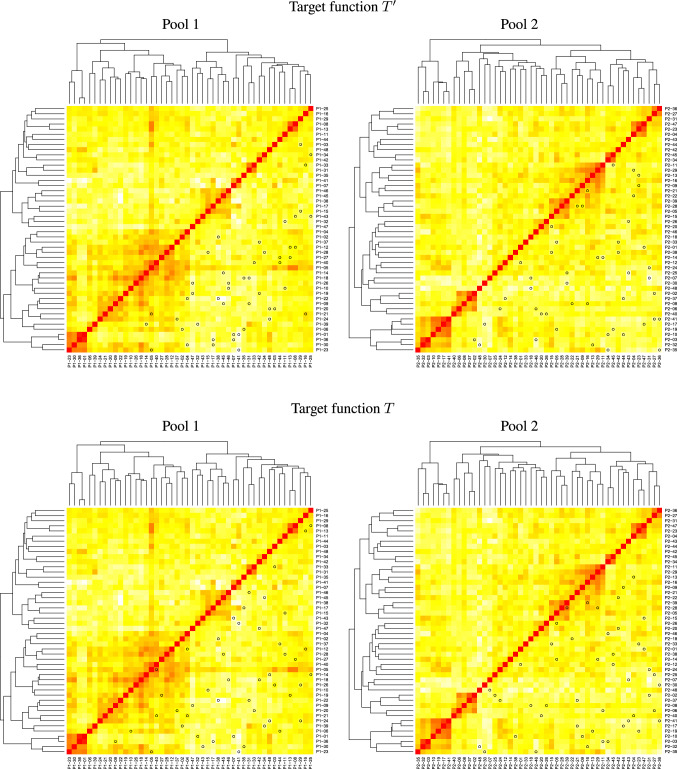
Heatmaps of the MRD (red: $\text {MRD}=0$ and white: $\text {MRD}=1$) between the parental lines ordered by hierarchical clustering. The crosses in recombination step R1 identified as favorable by the target functions $T'$ (top) and *T* (bottom) in pool 1 (left) and pool 2 (right) are marked by black dots in the heatmaps

**Fig. 3:**
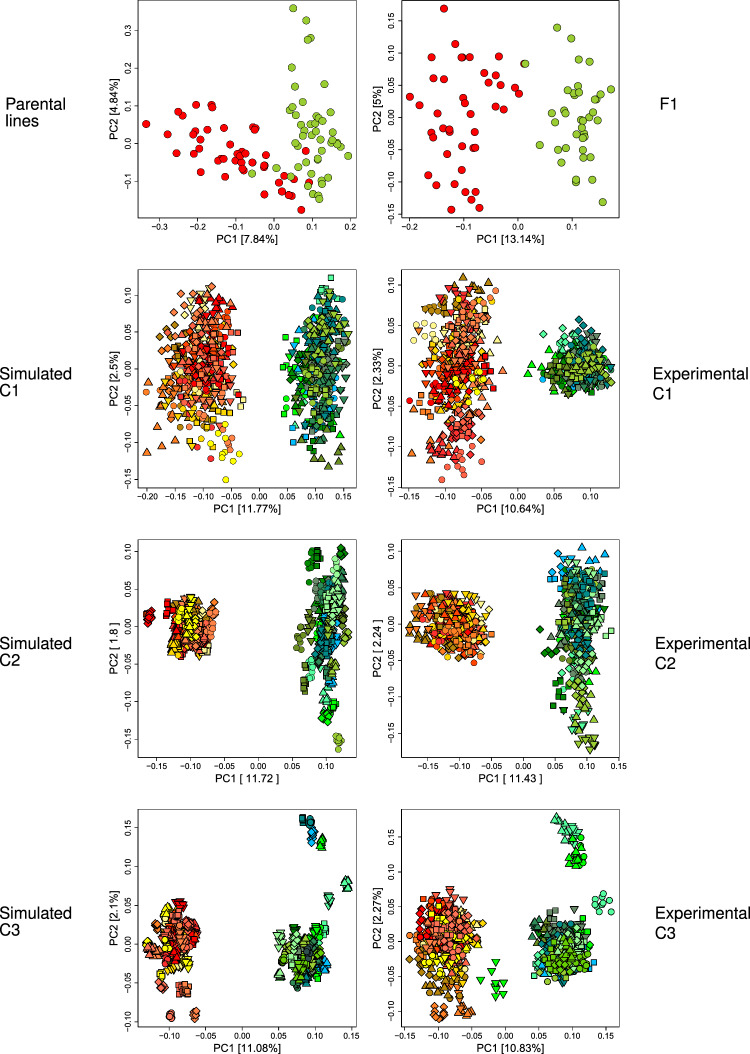
Principal coordinate plots of the two pools during the material development. In the first row, the parental lines and the F1 are shown. The latter three rows show cycles C1–C3. In the left column simulation, results were presented and, in the right column, the corresponding experimental data. Red symbols in generations P and F1, and yellow/red/brown coloring in generation C1–C3 were used for pool 1. Green symbols in generations P and F1, and green/blue coloring were used for pool 2. In generations C1–C3, different geometric shapes were used for genotypes that belong to different families

The parental inbreds of pool 1 showed two major clusters in the hierarchical cluster analysis (Fig. 2). Within the first cluster, four genotypes were closely related. The remaining genotypes belonged to one largely related group. In pool 2, there was a more fine-grained structure with several smaller groups of related lines. In the principal coordinate plot, the two sets of parental lines were partially separated with respect to the first principal coordinate; however, considerable parts of the two pools were overlapping in the principal coordinate plot (Fig. 3), and a clear separation of the pools was not observed.

The crosses of parental lines identified as favorable by the target functions *T* and $T'$ were determined (Fig. 2). The crosses identified by *T* were simulated and visualized in a principal coordinate plot (Fig. 3). These crosses were carried out experimentally. Due to the homozygosity of the parental lines, the experimental F1 plants had the same genotype and visualizations in the principal coordinate plots as the simulated ones. In F1, the two pools were separated by the first principal coordinate (Fig. 3). The two pools were located side by side, but a clear gap between the two pools was not yet visible.

The simulation of the C1 genotypes resulted in a clear separation of the two pools and a gap between them with respect to the first principal coordinate (Fig. 3). The gap had roughly the same extension on the first principal coordinate than the smaller of the two pools. The experimental C1 genotypes showed a gap between the pools that were not as big as is the simulations; nevertheless, the two germplasm pools were now clearly separated. The shape of the cloud of points in the principal coordinate plot was in accordance with the simulations for one of the two pools. For the second pool, the extension with respect to the second principal coordinate was smaller in the experimental data than in the simulated data.

Simulating the cycle C2 resulted in a clear separation of the two pools with a large gap between them with respect to the first principal coordinate (Fig. 3). The principal coordinate plots of the experimental C2 and C3 pools resembled closely that of the simulated ones.

Due to the stochastic nature of simulating meiosis of heterozygous genotypes, the simulation results presented in Fig. 3 are the outcomes of particular simulation runs, and different simulation runs will result in different outcomes. However, replications of the simulations showed outcomes that were to a large extent similar to the presented ones (results not shown).

The gene diversity in each of the two parental populations was 0.26. The crossing steps did change the gene diversity only marginally, so that in C3, it was still 0.26 in both populations. The average MRD between all genotypes of one pool with all genotypes of the other pool was 0.33, 0.32, 0.19, 0.34, and 0.28 in generations P, F1, C1, C2, and C3, respectively.

**Fig. 4:**
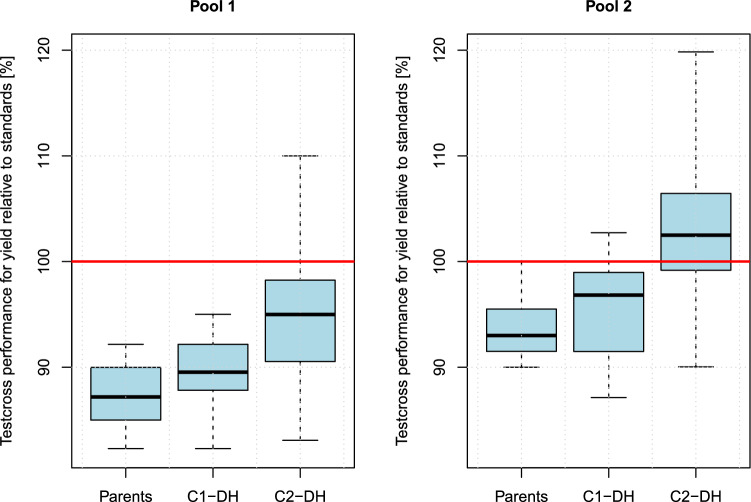
Relative testcross performance for yield for the parental lines and doubled haploid lines derived from cycles C1 and C2 (C1-DH and C2-DH). 100% is the mean performance of the three standards Ludger, DK Excited, and LG Aviron

The median of the testcross performance increased about 10% relative to the standards in both pools from the parental lines to the C2-DH lines (Fig. 4). Hence, the employed crossing scheme increased the relative testcross performance on average about 5% per crossing cycle.

## Discussion

### Methodology

The selection of crossing partners for recombination steps R1–R4 (Fig. 1) was accomplished by using the marker genotypes of the material from the previous generation and simulating alternative crossing strategies. The effect of the alternative decisions was evaluated visually by assessing the divergence of the pools in principal coordinate plots. This procedure did result in the desired separation of the two pools (Fig. 3) and in an increase in testcross performance (Fig. 4). However, we did not carry out a systematic evaluation of the effects of alternative selection strategies on pool separation and testcross performance.

We see the research need to do such a systematic evaluation of the crossing strategies presented here and plan to carry out a simulation study on this topic. We expect that such a simulation study will result in general guidelines on pool separation, which the ad hoc decisions of the case study presented here cannot yet provide. Nevertheless, from the proof of concept presented here, we conclude that planned pool separation in a pre-breeding program can be accomplished and that it is accompanied by an increase in testcross performance.

### Recombination R1

Our algorithm to determine the crosses for recombination R1 starts with identifying the best cross according to a target function and then adds subsequently favorable crosses under the restriction that each parental line is exactly used in two crosses.

**Fig. 5:**
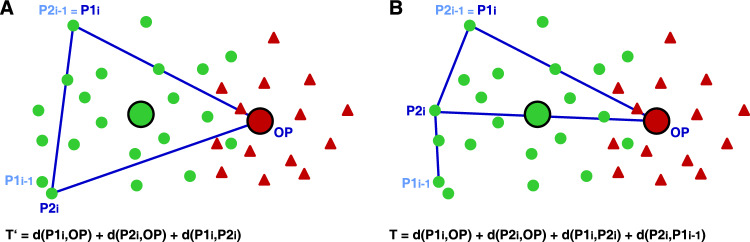
Illustration of the target functions $T'$ and *T* for selecting crosses in recombination R1. The lines of pool 1 are green, and the lines of pool 2 are red. P1_i-1_ and P2_i-1_ (light blue) are the parental lines of the $i-1$th cross. P1_i_ and P2_i_ (blue) are the parental lines of the *i*th cross, with $\text {P2}_{i-1} \equiv \text {P1}_{i}$. This illustration is a simplification in two respects: (1) It is using a two-dimensional space with an euclidean distance, whereas for the actual calculations, the MRD in a multi-dimensional space was used. (2) The opposite pool is represented by its centroid, whereas for the actual calculations, the average distance of a line to all lines of the opposite pool was used

The target function $T'$ (Eq. 1) favors crosses for which the distances of the two parental lines to the opposite pool are large, and in addition, the distance between the parental lines is large (Fig. 5A). If such a cross is considered stand-alone, then it might have optimum properties from a quantitative genetic point of view, as in such a cross, two favorable genotypes that are genetically different are recombined. But as we determined the crosses to be carried out sequentially, and the first parent of a subsequent cross is always the second parent of the previous cross, the target function *T* often added similar crosses to the list of crosses to be carried out. This is illustrated in Fig. 5A. The lines $\text {P1}_{i-1}$ and $\text {P2}_{i}$ are similar, and therefore, the crosses $i-1$ and *i* are similar. In Fig. 2, this property of *T* results in many pairs of crosses where one line is crossed with two other lines that are grouped together closely by the hierarchical clustering. Carrying out similar crosses for generating the next generation is not optimal with respect to generate a large segregation variance in subsequent generations.

The target function *T* extends the target function $T'$ by the distance between the lines that are not common in two subsequent crosses $\text {P1}_{i-1}$ and $\text {P2}_{i}$. This ensures that a given line $P1_{i}$ is crossed with two lines $\text {P1}_{i-1}$ and $\text {P2}_{i}$, which are not closely related. The effect is illustrated in Fig. 5B and is verified in Fig. 2. We suppose that a considerable part of the success in pool separation and the increase in testcross performance of our crossing program is caused by the high segregation variance introduced by using the target function *T*.

### Recombination R2

The target function *T* used in recombination R1 had the goal to preferably recombine lines that are distant from the opposite pool. When employed repeatedly in simulations (results not shown), the extremes of the pools separated quickly. However, crossing of genotypes that were close to the opposite pool resulted in progenies of which some were further away from the opposite pool than their parents, but still in close proximity to the opposite pool.

**Fig. 6:**
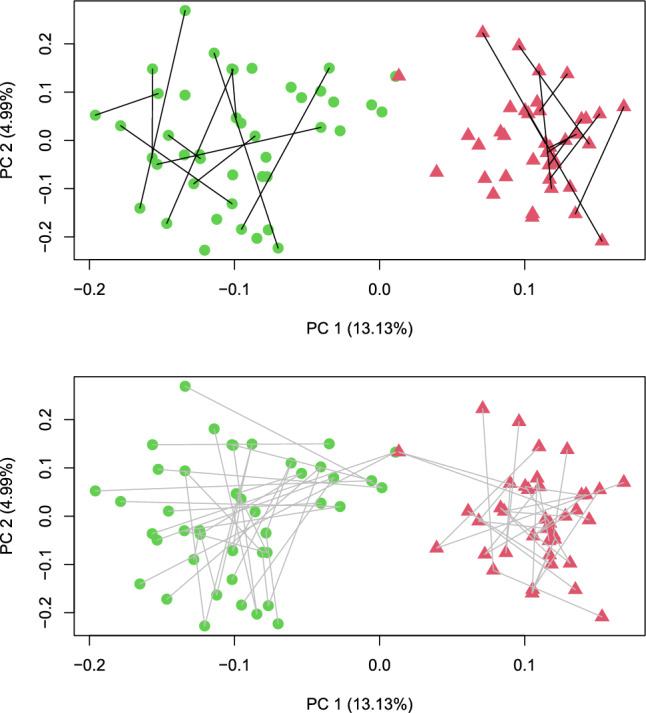
Crosses of the F1 genotypes. The black lines (top) connect F1 genotypes that were selected as crossing partners of crosses of type I (“best x best”). The gray lines (bottom) connect genotypes selected as parents of crosses of type II (“improve the worst”)

Starting with recombination R2, we switched from the “best $\times$ best” strategy of target function *T* to a strategy where only 1/3 of the crosses were “best $\times$ best” crosses. The target function $T_b$ still identifies “best $\times$ best” but compared with *T*, the importance of the distances between the two parents was reduced, and moreover, a line was used at maximum in one “best $\times$ best” cross. The remaining two-thirds of the crosses were determined with the target function $T_w$ that chooses one parental line that is far away from the opposite pool and one line that is close to the opposite pool. The effect of using $T_b$ for one-third of the crosses and $T_w$ for the remaining two-thirds to “improve the worst” is illustrated in Fig. 6. We attribute the observation that starting from cycle C1, there is a clear separation between the pools visible in the principal coordinate plot (Fig. 3) to the effect of target function $T_w$.

### Recombinations R3 and R4

Starting with recombination R3, we integrated genomic predictions of testcross performance in planning the crosses as we had testcross data from the parental lines available. The focus of our material development was the pool separation under the side conditions to maintain genetic variety of the parental lines and improve the testcross performance. Therefore, we did not rely entirely on strong selection for the testcross performance, but instead combined it with our strategy of using the target functions $T_b$ and $T_w$. Our combined strategy of employing $T_b$ and $T_w$ on the family level targeted at maintaining the pool separation and minimizing the loss of genetic variation, as each family is used for two crosses. The genomic predictions to determine which genotypes of a family should be crossed targeted at making use of the within family variation for improving the testcross performance.

### Application in breeding programs

The material that we developed in this project is ready to be used as the starting material for developing the heterotic pools of a hybrid breeding program. Compared with the parental lines, the C3 material showed a clear separation of the pools (Fig. 3), and the C1-DH and C2-DH lines showed an improved testcross performance (Fig. 4) of about 5% relative yield per crossing generation.

We consider it as a reasonable guess, that one cycle of RRS results in a yield increase of 5%, which is similar to the yield increase that we observed for one generation of planned crosses. However, employing a pre-breeding strategy has several advantages compared to a direct start of testcross based selection: It is faster and cheaper, and it maintains a greater genetic diversity in the breeding material.

A cycle of an RRS program could consist, for example, of the following steps: (1) carrying out crosses, (2) generating DH lines, (3) generating testcross seeds, and (4) two selection steps for testcross performance. Then, step (1) of the next breeding cycle would follow. For these steps, a 5-year time span might be required. In contrast, for carrying out one generation of planned crosses, only 1 year is required. If we consider the improvement of the C2-DH lines that we experimentally observed, then this could be the result of two RRS cycles that take 10 years. In contrast, carrying out planned crosses takes only 2 years. From a breeder’s point of view, this is a tremendous advantage of the pre-breeding approach, that might even determine the competitiveness of the breeding program.

Further, the selection intensity in our program was much lower than in a testcross-based selection program where typical high selection intensities are employed. As a consequence, our material in cycle C3 is assumed to cover much more of the genetic diversity of the founder lines, than after 4 cycles of testcross-based selection. Hence, a larger long-term selection response can be expected. We conclude that pre-breeding efforts like described in this study can help to efficiently start a hybrid breeding program.

## Data Availability

The data are available from the authors on request.
